# Colorectal Cancer Is Associated with a Deficiency of Lipoxin A_4_, an Endogenous Anti-inflammatory Mediator

**DOI:** 10.7150/jca.32456

**Published:** 2019-08-20

**Authors:** Haojing Liu, Ji Zeng, Wei Huang, Qiang Xu, Duyun Ye, Rui Sun, Dongxin Zhang

**Affiliations:** 1 Department of Internal Medicine, Wuhan Fourth Hospital; Puai Hospital, Tongji Medical College, Huazhong University of Science and Technology, Wuhan 430033, People's Republic of China; 2 Department of Clinical Laboratory, Wuhan Fourth Hospital; Puai Hospital, Tongji Medical College, Huazhong University of Science and Technology, Wuhan 430033, People's Republic of China; 3 Department of Pathophysiology, Tongji Medical College, Huazhong University of Science and Technology, Wuhan 430030, People's Republic of China; 4 Department of Oncology, Wuhan Fourth Hospital; Puai Hospital, Tongji Medical College, Huazhong University of Science and Technology, Wuhan 430033, People's Republic of China

**Keywords:** colorectal cancer, immune microenvironment, inflammation, lipoxin A_4_, subcutaneous xenograft

## Abstract

Unresolved inflammation, due to insufficient production of proresolving anti-inflammatory lipid mediators, can lead to tumorigenesis. Among these mediators, lipoxin A_4_ (LXA_4_) has potent anti-carcinogenic properties, and may serve as key target for modulating inflammation-associated cancer like colorectal cancer. The purpose of present study was to clarify the roles of LXA_4_ in colorectal cancer. We investigated the effects and underlying mechanisms of LXA_4_ in colorectal cancer and its relationship with tumor-associated inflammation and immune microenvironment by employing clinical samples and mouse colorectal cancer cell line CT26-bearing tumor model as well as colorectal cancer cells. It was found that colorectal cancer is associated with dysregulation of immune microenvironment and deficiency of LXA_4_ that could play different roles at different stages of tumor growth: inhibiting early but promoting late tumor growth. Analysis of peripheral immune cells in subcutaneous xenograft mice model disclosed that early LXA_4_ treatment induced lymphocytes and inhibited neutrophils and monocytes, while late LXA_4_ treatment induced neutrophils but inhibited lymphocytes. Detailed analysis of tumor microenvironment revealed that early LXA_4_ treatment could inhibit inflammatory mediators expressions and leukocytes infiltration into tumor. Furthermore, LXA_4_ could suppress the expressions of p-ERK, p-P38 and NF-κB in subcutaneous xenograft. Additionally, LXA_4_ could inhibit the proliferation and migration of colorectal cancer cells, and, meanwhile, inhibit the proliferation and migration of colorectal cancer cells stimulated by activated macrophage-conditioned media. These findings suggest that colorectal cancer is associated with a deficiency of LXA_4_ that could suppress colorectal cancer via modulating tumor-associated inflammation and immune microenvironment as well as inhibiting colorectal cancer cell development.

## Introduction

Inflammation plays decisive roles at different stages of tumor development, including initiation, promotion, malignant conversion, invasion and metastasis^1^. It has become evident that an inflammatory microenvironment is an essential component of tumor, since immune cells that infiltrate tumors engage in an extensive and dynamic crosstalk with cancer cells by inflammatory mediators^1^. It's well established that inflammatory bowel disease is an important risk factor for the development of colon cancer, and even colorectal tumors that are not associated with clinically detectable inflammatory bowel disease display robust inflammatory infiltration and increased expression of pro- inflammatory cytokines^2^.

Unresolved inflammation, due to insufficient production of proresolving anti-inflammatory lipid mediators, can lead to an increased risk of tumorigenesis 3. Various bioactive lipids, particularly those formed by cyclooxygenase (COX) and lipoxygenase (LOX) enzymes, have been well established as therapeutic targets for many cancers like colon carcinogenesis^3^, ^4^. Emerging studies suggest a role of anti-inflammatory bioactive lipid mediators in the resolution of inflammation, and among these proresolving mediators, LXA_4_ have potent anti-inflammatory and anti-carcinogenic properties^3^. In fact, LXA_4_ has been extensively studied for its anti-inflammatory and inflammatory pro-resolving effect in intestinal inflammation^5^. Of interest, long-term use of non-steroidal anti- inflammatory drug aspirin reduces the risk of several cancers like colorectal cancer^6^. Aspirin could trigger the endogenous formation of carbon-15 epimeric LXA_4_, namely aspirin-triggered LXA_4_^7^, that may account for some of the bioactivity profile of aspirin in colorectal cancer. Moreover, our previous studies confirmed that LXA_4_ could suppress hepatocellular carcinoma via remodeling tumor immune microenvironment and angiogenesis^8^, ^9^. We also showed that LXA_4_ could inhibit tumorigenesis via remodeling macrophage polarization^10^. Hence, LXA_4_ may serve as a key target for regulation of inflammation-associated cancer like colorectal cancer.

Currently, it's well accepted that dual anti-inflammatory and proresolving mediator LXA_4_ is considered as “braking signals” of inflammation. Moreover, LXA_4_ has been confirmed to have potent anti-inflammatory and anti-carcinogenic properties from other reports and our studies. However, whether LXA_4_ deficiency may result in colorectal cancer development is not known, and whether LXA_4_ play important roles in inhibiting colorectal cancer development remains to be revealed. Also, the regulation of colorectal cancer and associated inflammation by LXA_4_ remains unclear. Thus, the purpose of the present study was to solve these unknown questions.

## Materials and Methods

### Reagents and antibodies

3-[4,5-dimethylthiazol-2-yl]-2,5-diphenyltetrazolium bromide (MTT) and LPS (Escherichia coli serotype O127:B8) were purchased from Sigma Aldrich (Allentown, PA, USA). LXA_4_ (Cayman Chemical, USA) was stored at -80°C until being diluted in sterile 0.9% saline or cell culture medium immediately before use. Anti NF-κB, ALOX12, ALOX15, ALOX15B and Lamin B antibodies were purchased from Santa Cruz Biotechnology (Santa Cruz, CA, USA). Mouse ALOX5 monoclonal antibody (BD Biosciences, USA) was used for western blotting, and rabbit ALOX5 polyclonal antibody (Cayman Chemical, USA) was used for immunohistochemistry (IHC). Anti p-ERK, ERK, p-P38 and P38 antibodies were from Cell Signaling Technology (Danvers, MA, USA). Anti CD45 and CD68 antibodies were from Proteintech Group, Inc. (Wuhan, China). RIPA Lysis Buffer and Nuclear and Cytoplasmic Protein Extraction Kit were from Beyotime Institute of Biotechnology (Shanghai, China). BCA protein assay kit was from Pierce (Rockford, IL, USA). Apoptosis detection kit (Annexin V and PI) was purchased from BD Pharmingen.

### Patient samples

Human colorectal cancer tissue and surrounding tissue were obtained from patients with well-differentiated colorectal cancer after gastrointestinal surgery in Puai Hospital, Tongji Medical College, Huazhong University of Science and Technology. Blood samples were obtained from 24 patients: 12 patients with well-differentiated colorectal cancer, and 12 healthy controls were originally selected with characteristics similar to those presented by colorectal cancer patients-including body mass index, age and so on, and controls were excluded if they showed any signs of illness or were currently on medication. Both blood and colorectal cancer samplings were obtained from diagnosed patients after informed consent and approved by the Ethical Committee of Puai Hospital, Tongji Medical College, Huazhong University of Science and Technology in accordance with the Declaration of Helsinki.

### Mice model and experimental protocol

BALB/c male mice (6-8 week old, weighing 18-22g) were from the Experimental Animal Center of Tongji Medical College, Huazhong University of Science and Technology. Animals were housed individually in plastic cages with wood chips as bedding, under pathogen-free conditions, at 20-25°C, and 12L:12D. Mice were fed a standard laboratory diet and water *ad libitum*. All animal work was conducted according to the Guide for the Care and Use of Laboratory Animals of the National Institutes of Health. All studies involving mice were approved by Animal Care and Use Committee of Huazhong University of Science and Technology.

#### Experimental protocol 1: LXA_4_ administration at early subcutaneous xenograft

To establish subcutaneous xenograft mice model, BALB/c background colon cancer cell line CT26 was *s.c.* injected into the right flank of BALB/c mice to generate individual tumors. Mice were randomly divided into control group, CT26-bearing group and CT26-bearing+LXA_4_ group. Mice in control group and CT26-bearing group were injected *i.p.* with vehicle (saline), while mice in CT26-bearing+LXA_4_ group were injected *i.p.* with LXA_4_ (10μg/kg) on day 7 after inoculation. Subcutaneous tumor growth was monitored by measuring the length (L) and width (W) of tumors using vernier calipers, and the volume (V) of the tumor was calculated by formula V=(L*W^2^)/2. At the time of autopsy on day 14 after inoculation, tumors were dissected and weighed.

#### Experimental protocol 2: LXA_4_ administration at late subcutaneous xenograft

Subcutaneous xenograft mice model was established according to above method, and mice were then randomly divided into control group, CT26-bearing group and CT26-bearing+LXA_4_ group. Mice in control group and CT26-bearing group were injected *i.p.* with vehicle (saline) on day 15 after inoculation, while mice in CT26-bearing + LXA_4_ group were injected *i.p.* with LXA_4_ (10μg/kg). Subcutaneous tumor growth was monitored by measuring the V of tumors. At the time of autopsy on day 22 after inoculation, tumors were dissected and weighed.

### Cell culture and treatment

Human colorectal cancer cell line SW480 was obtained from American Type Culture Collection (USA). Cells were cultured at 37°C in a humidified atmosphere in L15 medium supplemented with 10% heat-inactivated fetal bovine serum (FBS), 100 U/ml penicillin G, and 100 U/ml streptomycin. SW480 cells were plated in 96-well plates, 6-well plates or 12-well plates and treated with different indicated conditions for 24 h for proliferation, migration and apoptosis experiments respectively.

### Isolation of human peripheral blood monocyte (PBM) and preparation of activated macrophage-conditioned media (ACM)

Peripheral venous blood from healthy donors was diluted in phosphate buffered saline (PBS) and subjected to one-step density centrifugation over Ficoll (GE Health, USA). The interface containing mononuclear cells was collected, washed in PBS, and resuspended in fresh 1640 culture medium supplemented with 10% heat-inactivated FBS, 25 mmol/L HEPES, 100 U/ml penicillin G and 100 U/ml streptomycin at 10^6^ cells/ml. The monocyte/ macrophage population was obtained by plastic- plating of peripheral blood mononuclear cells (4 hours at 37°C) and subsequent removal of nonadherent T and B cells. Fresh medium was added to the adherent cells, which were then incubated at 37°C in 5% CO_2_ before use in further studies. For the preparation of macrophage ACM, PBMs were treated for 1h with 1μg/ml LPS. After washed twice with PBS, cells were kept in culture media for 24 h, and then ACM was collected, filtered through a syringe filter (0.45μm pore size, Fisher Scientific) and added to SW480 cells.

### Mice leukocyte differential count

Before the time of autopsy, peripheral blood was obtained from mouse eyes and placed in EDTA-K_2_ anticoagulating solution. Peripheral blood leukocytes were classified and counted by whole blood cell counter (Sysmex XE5000, Japan) based on the principle of flow cytometry.

### Cell proliferation assay

Cell proliferation was determined by a colorimetric method based upon metabolic reduction of the soluble yellow tetrazolium dye MTT to its insoluble purple formazan. Approximately 5000 cells/well were grown in 96-well plates overnight in 200μl of culture medium, and then incubated under indicated conditions for 24 h. Each well was added with 20μl MTT (0.5 mg/ml) and incubated for 4 h before supernatant was removed. After plate was placed at 37 °C for 15min in 150μl DMSO, the absorbency was measured with a micro ELISA reader (Amersham Biosciences, USA) at a wavelength of 492 nm.

### Cell migration assay

Wound healing assay was employed to assess cell migration. Briefly, SW480 cells were grown on 6-well plates in culture medium. After formation of a confluent monolayer, straight wounds were created using a sterile pipette tip and cells were treated with indicated conditions for 24 h. Microscopic photographs were taken at 0 and 24 h respectively. Relative migration ratio=migration distance/wound width.

### Cell apoptosis detection by flow cytometry

SW480 cells were grown on 12-well plates overnight, and then treated with indicated conditions for 24 h. Cells were washed with PBS before apoptosis detection. Apoptotic cells were detected by flow cytometry after double staining with Annexin V and PI.

### Immunohistochemistry (IHC)

Cancer tissues were fixed in 10% neutral buffered formaldehyde solution. After dehydration procedures, the samples were blocked in paraffin. For assessment of different tumor protein expression, regular IHC assay was performed. Briefly, 4μm paraffin sections of tumor specimens were cut, and after deparafinization, antigen retrieval was performed in sodium citrate solution (pH 6.0). Sections were incubated with different primary antibody at 4°C overnight. Sections were then washed and incubated with secondary antibody for 45 min at room temperature. The sections were subsequently incubated with 3,3'-diaminobenzidine tetrahydrochloride (DAB) substrate, lightly counterstained with hematoxylin, dehydrated, and mounted. IHC staining was observed under light microscopy (Olympus BX60). The magnification for the IHC was 200 times.

### Western blotting

Total and nuclear proteins were extracted using RIPA Lysis Buffer and Nuclear and Cytoplasmic Protein Extraction Kit, respectively. Protein concentrations were determined using a BCA protein assay kit. Equal amounts of protein (40μg) were then subjected to 12% SDS- PAGE and transferred to PVDF membranes (Millipore). Different primary antibodies were incubated with the membranes overnight at 4°C. Lamin B served as internal control. The bound antibody was detected by an enhanced chemiluminescence kit (Millipore) on X-ray film.

### Enzyme-linked immunosorbent assay (ELISA)

The concentrations of human IL1B, IL6, CXCL8, TNFA, CCL2 and IL10 and mouse IL1B, IL6, CXCL2, TNFA, CCL2 and IL10 in the serum and tumor tissue were detected using ELISA kits (R&D Systems, USA) according to the manufacturer's instructions.

### Determination of LXA_4_ and leukotriene B_4_ (LTB_4_)

Before measurement of tumor LXA_4_ and LTB_4_ concentrations, tissue homogenization was performed to extract the tissue contents in tumor. Briefly, tumor samples were homogenized in mild homogenate buffer containing a cocktail enzyme inhibitor by using the electric mechanical homogenizer. Twenty minutes later, homogenate samples were centrifuged at 3000 rpm at 4°C for 20 min. Supernatants were then collected and stored at -80°C until determination. Levels of LXA_4_ and LTB_4_ in the serum and tumor were assessed by assay kit according to manufacturers' protocols (Neogen Corp.; Cayman Chemical).

### Statistical analysis

All statistical analyses were done using the SPSS 19.0 software. The results were expressed as means±S.E.M of multiple independent experiments. The means of different groups were compared by employing either Student's t-test or one-way ANOVA followed by S-N-K post-hoc test. A value of p<0.05 was considered significant.

## Results

### Expressions of LXA_4_ in colorectal cancer patients

**Figure 1:**
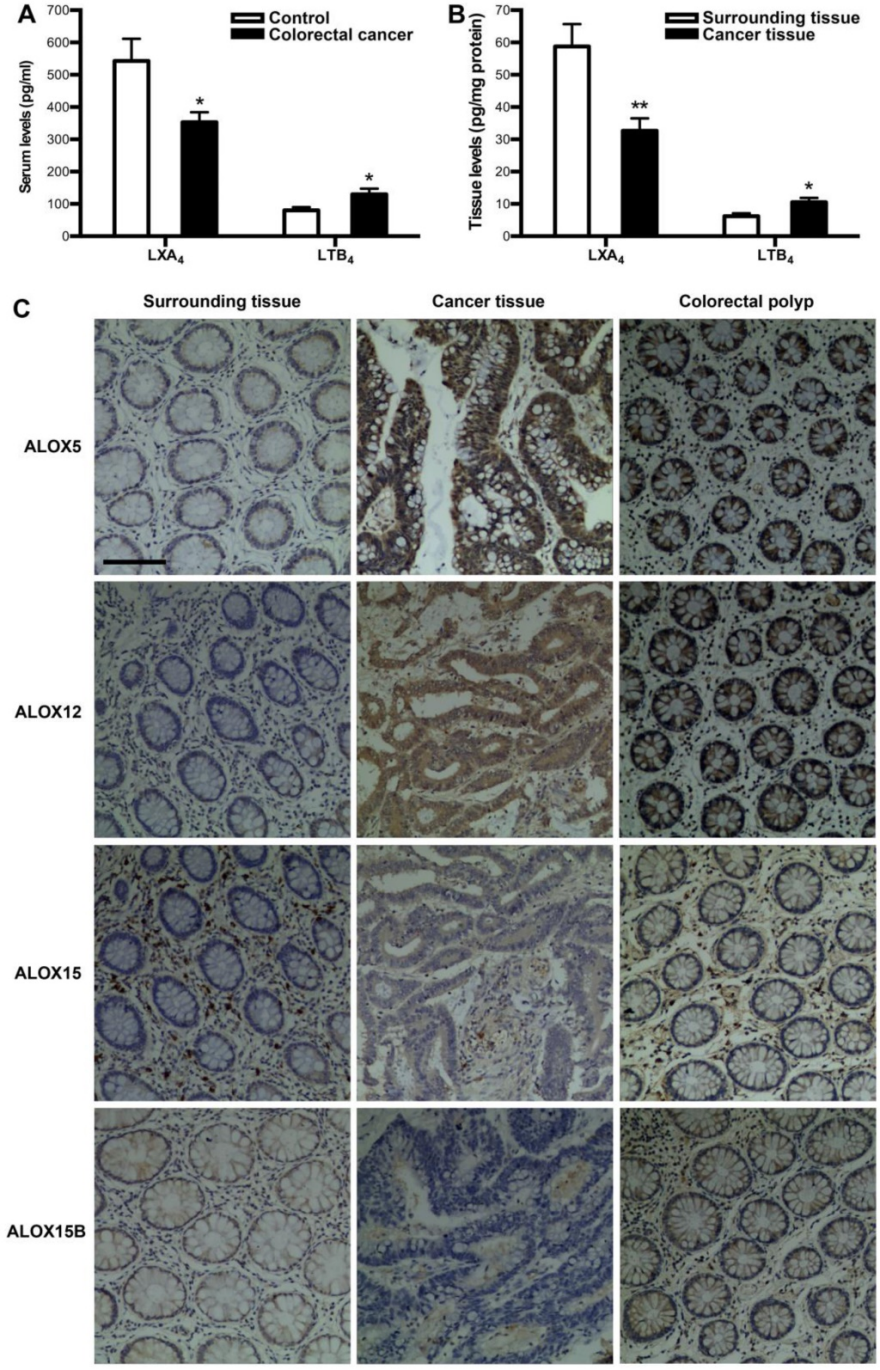
** The expressions of LXA_4_ in colorectal cancer patients.** (A) Comparison of serum LXA_4_ and LTB_4_ levels between colorectal cancer patients and healthy controls. (B) Comparison of LXA_4_ and LTB_4_ levels between tumor tissue and surrounding tissue of colorectal cancer patients. Results are expressed as means±SEM (n=12 in each group). *p<0.05 and **p<0.01 versus control group, two-tailed Student's t-test. (C) The expressions of LXA_4_-synthesizing enzymes in colorectal cancer patients. IHC analysis of ALOX5, ALOX12, ALOX15 and ALOX15B in tumor tissue, surrounding tissue and colorectal polyp was shown. Original magnification, 200; bar=100μm

To verify whether colorectal cancer is associated with a deficiency of LXA_4_, we measured the levels of LXA_4_ in serum and tumor of colorectal cancer patients. Meanwhile, we detected the levels of proinflammatory LTB_4_, since it's the other metabolite of LXA_4_ biosynthesis key enzyme ALOX5. As shown in Fig. 1A, serum LXA_4_ levels of colorectal cancer patients were decreased, compared with healthy controls. In line with this, LXA_4_ levels in tumor were significantly lower than those in surrounding tissue (Fig. 1B). In contrast, LTB_4_ were upregulated in both serum and tumor of colorectal cancer patients (Fig. 1A and 1B). LXA_4_ is endogenously generated via transcellular biosynthesis from arachidonic acid with the sequential catalysis of ALOX15 and ALOX5 or ALOX5 and ALOX12, and ALOX15 exists in two isoforms: ALOX15 and ALOX15B^7^, ^11^. To confirm whether LXA_4_-synthesizing enzymes are dysregulated in colorectal cancer, we detected the expressions of ALOX5, ALOX12, ALOX15 and ALOX15B in colorectal cancer tissue. As shown by IHC staining (Fig. 1C), the expressions of ALOX15 and ALOX15B were strikingly down-regulated, while ALOX5 and ALOX12 were strikingly up-regulated in tumor, compared with those in surrounding tissue. Furthermore, we detected the expressions of ALOX5, ALOX12, ALOX15 and ALOX15B in colorectal polyp. As a result, ALOX5 and ALOX12 were strikingly up-regulated in colorectal polyp, compared with those in colorectal cancer surrounding tissue, while there were no significant change in the expressions of ALOX15 and ALOX15B (Fig. 1C). These clinical results suggest that colorectal cancer is correlated with low levels of LXA_4_ while high levels of LTB_4_, and the dysregulation of LXA_4_-synthesizing enzymes may be the reason for LXA_4_ deficiency in colorectal cancer.

### Tumor-associated inflammation and immunity in colorectal cancer patients

**Figure 2:**
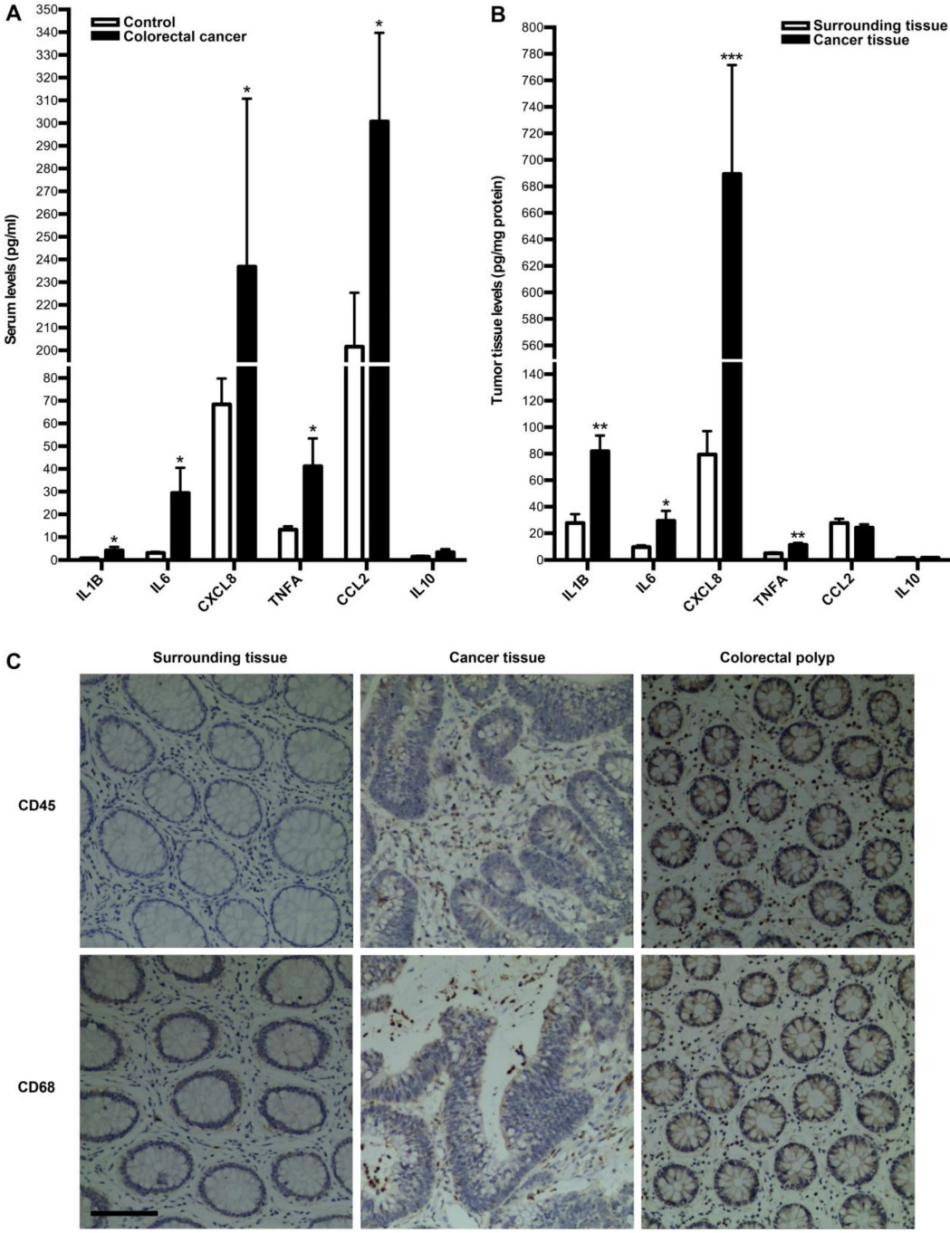
** Inflammatory cytokines expressions and leukocytes infiltration into tumor in colorectal cancer patients.** (A) Comparison of serum inflammatory cytokines levels between colorectal cancer patients and healthy controls. (B) Comparison of inflammatory cytokines levels between tumor tissue and surrounding tissue. Results are expressed as means±SEM (n=12 in each group). *p<0.05, **p<0.01 and ***p<0.001 versus control group, two-tailed Student's t-test. (C) Leukocyte and macrophage infiltration into tumor tissue, surrounding tissue and colorectal polyp. IHC analysis by using CD45 and CD68 antibodies in tumor tissue, surrounding tissue and colorectal polyp was shown. Original magnification, 200; bar=100μm

Next, we investigated whether tumor immune microenvironment is dysregulated in colorectal cancer by employing clinical specimens from colorectal cancer patients. We first measured the expressions of inflammatory cytokines in serum and tumor of colorectal cancer patients. As a result, the serum levels of IL1B, IL6, CXCL8, TNFA and CCL2 were significantly up-regulated in colorectal cancer patients, compared with those in healthy controls, while there is no difference for IL10 between two groups (Fig. 2A). In line with this, the levels of IL1B, IL6, CXCL8 and TNFA in tumor were significantly higher than those in surrounding tissue, although there is no difference for IL10 and CCL2 between two groups (Fig. 2B). Moreover, we focused on immune cells in tumor microenvironment. IHC of CD45 and CD68 were employed to assess leucocytes and macrophages infiltration into colorectal tumor. As shown in Fig. 2C, there was much more obvious infiltration of both immune cells into tumor. Additionally, there was much more obvious infiltration of both immune cells into colorectal polyp (Fig. 2C). These results suggest that colorectal cancer is correlated with the dysregulation of tumor immune microenvironment.

### Effects of LXA_4_ on subcutaneous xenograft in mice

**Figure 3:**
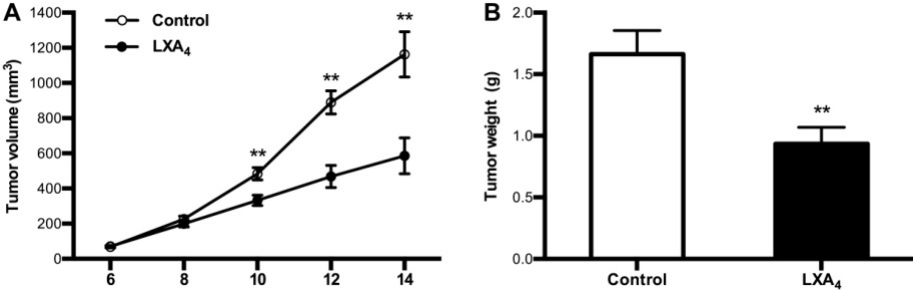
** The inhibition of LXA_4_ on early subcutaneous xenograft in mice.** Mouse colorectal cancer cell line CT26 was used to prepare subcutaneous xenograft. Tumor-bearing mice were injected *i.p.* with LXA_4_ on day 7 after CT26 inoculation, and peripheral tumor volume was measured every other day. At the time of autopsy on day 14, tumors were dissected and weighed. (A) Comparison of tumor volume between control and LXA_4_ group. (B) Comparison of tumor weight between control and LXA_4_ group. Results are expressed as means±SEM (n=7 mice in each group). **p<0.01 versus control group, two-tailed Student's t-test

Given LXA_4_ deficiency in colorectal cancer, we next determined whether LXA_4_ could inhibit tumor growth *in vivo* by employing mouse colorectal cancer cell line CT26-bearing mice model. Unexpectedly, LXA_4_ could prompt tumor growth as shown by volume measurement when mice were treated with LXA_4_ at late stage (Fig. S1A). However, LXA_4_ could significantly inhibit tumor growth when mice were treated with LXA_4_ at early stage (Fig. 3A). In line with this, tumor weight of LXA_4_ treatment group was increased at late stage (Fig. S1B), while tumor weight of LXA_4_ treatment group was significantly decreased at early stage (Fig. 3B). These results suggest that LXA_4_ inhibits early while promotes late subcutaneous xenograft growth in mice model.

### Modulation of LXA_4_ on tumor-associated inflammation and immunity in subcutaneous xenograft model

**Figure 4:**
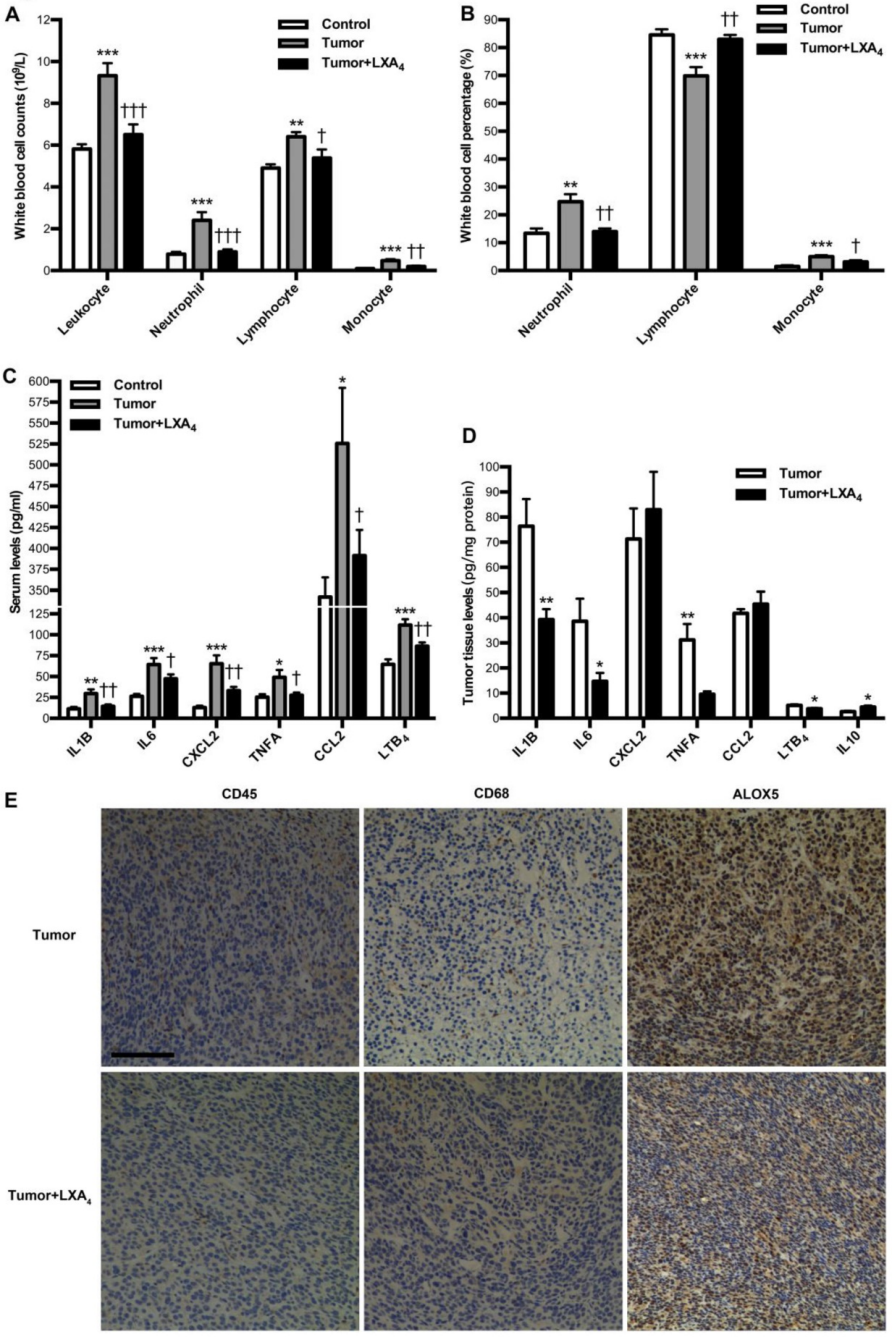
** Effects of LXA_4_ on tumor-associated inflammation and immunity in subcutaneous xenograft model.** Subcutaneous xenograft was prepared, and mice were injected *i.p.* with LXA_4_ on day 7 after inoculation. At the time of autopsy on day 14, peripheral blood was collected from mouse eyes, and peripheral leukocytes were classified and counted by whole blood cell counter. The concentrations of inflammatory mediators in serum and tumor were detected by using ELISA kit. (A) Comparison of peripheral blood leukocyte counts between control, tumor and LXA_4_ group. (B) Comparison of peripheral blood leukocyte classification between control, tumor and LXA_4_ group. (C) Comparison of serum inflammatory mediators levels between control, tumor and LXA_4_ group. Results are expressed as means±SEM (n=7 mice in each group). *p<0.05, **p<0.01 and ***p<0.001 versus control group, ^†^P<0.05,^ ††^P<0.01 and ^†††^P<0.001 versus tumor group, one-way ANOVA with S-N-K posttest. (D) Comparison of inflammatory mediators levels in tumor between tumor and LXA_4_ group. Results are expressed as means±SEM (n=7 mice in each group). *p<0.05 and **p<0.01 versus control group, two-tailed Student's t-test. (E) The effects of LXA_4_ on leukocytes infiltration and ALOX5 expression in subcutaneous xenograft. Leukocytes and macrophages infiltration into tumor was performed by IHC analysis using CD45 and CD68 antibodies. Original magnification, 200; bar=100μm

We next investigated the influence of LXA_4_ on peripheral immune cells in subcutaneous xenograft model. At late stage of subcutaneous xenograft, peripheral leukocytes increased, while LXA_4_ could obviously inhibit their increase; Neutrophils and its percentage were higher than those in control, while LXA_4_ could further up-regulate neutrophil percentage of peripheral leukocytes; Lymphocytes increased while its percentage decreased compared to controls, and LXA_4_ could down-regulate lymphocytes and its percentage; Both counts and percentage of monocyte increased compared to controls, and LXA_4_ had on effects on monocyte (Fig. S2A and Fig. S2B). At early stage, peripheral leukocytes increased, while LXA_4_ could obviously inhibit their increase; Both counts and percentage of neutrophils were higher than controls, while LXA_4_ could down-regulate neutrophils and its percentage; Lymphocyte counts increased while its percentage decreased compared to controls, and LXA_4_ could down-regulate lymphocytes but up-regulate its percentage; Both counts and percentage of monocyte increased compared to controls, while LXA_4_ could down-regulate monocyte counts and its percentage (Fig. 4A and B). These results suggest that differential effects of LXA_4_ on subcutaneous xenograft may be explained by its modulation on peripheral immune cells. To further reveal the detailed mechanisms responsible for LXA_4_-mediated inhibition on early subcutaneous xenograft in mice model, we investigated the effects of LXA_4_ on inflammatory mediators expression and immune cells infiltration in subcutaneous xenograft. As shown in Fig. 4C, the serum levels of IL1B, IL6, CXCL2, TNFA, CCL2 and LTB_4_ were significantly higher than those in control, while LXA_4_ could obviously inhibit these pro-inflammatory mediators expression. In line with this, LXA_4_ treatment could obviously suppress the tumor levels of IL1B, IL6, TNFA and LTB_4_ while up-regulate IL10 expression, although it had no effects on CXCL2 and CCL2 (Fig. 4D). Moreover, we focused on immune cells infiltration into subcutaneous xenograft. As shown by the IHC results of CD45 and CD68 in Fig. 4E, there was obvious infiltration of leukocytes and macrophages into subcutaneous xenograft, while LXA_4_ could significantly inhibit their infiltration. Meanwhile, LXA_4_ could significantly inhibit ALOX5 expression in subcutaneous xenograft (Fig. 4E). These results suggest that LXA_4_ may inhibit early colorectal cancer by remodeling tumor immune microenvironment.

### Inhibition of LXA_4_ on MAPKs and NF-κB pathways in subcutaneous xenograft

**Figure 5:**
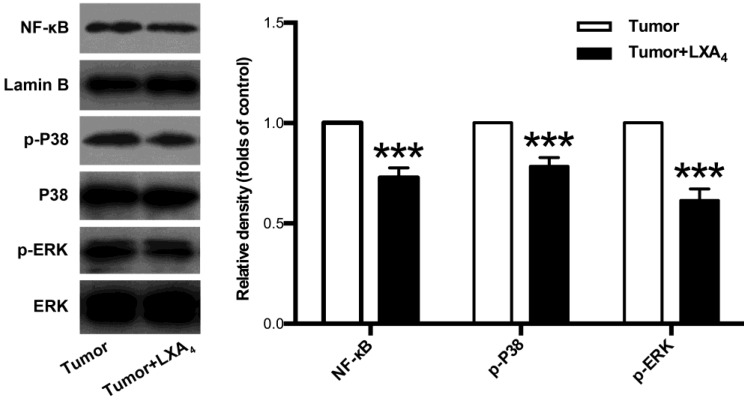
** The effects of LXA_4_ on NF-κB, P38 and ERK in subcutaneous xenograft.** Subcutaneous xenograft was prepared, and tumor-bearing mice were injected *i.p.* with LXA_4_ on day 7 after inoculation. At the time of autopsy on day 14, the expressions of NF-κB, p-P38 and p-ERK in tumor were detected by using western blotting. The histogram represents means±SEM of the densitometric scans for protein bands from n=7 mice, normalized by comparison with Lamin B, P38 and ERK and expressed as a percentage of tumor group. ***p<0.001 versus tumor group, two-tailed Student's t-test

Since MAPKs and NF-κB pathways are important signals involved in tumor development and tumor-associated inflammation^12^, ^13^, we next examined the effects of LXA_4_ on ERK, P38 and NF-κB in subcutaneous xenograft. As shown in Fig. 5, LXA_4_ could obviously suppress the expressions of p-ERK, p-P38 and NF-κB in subcutaneous xenograft. These findings suggest that LXA_4_ may inhibit colorectal cancer and tumor-associated inflammation via modulating ERK, p38 and NF-κB.

### Regulation of LXA_4_ on proliferation, apoptosis and migration of colorectal cancer cells* in vitro*

**Figure 6:**
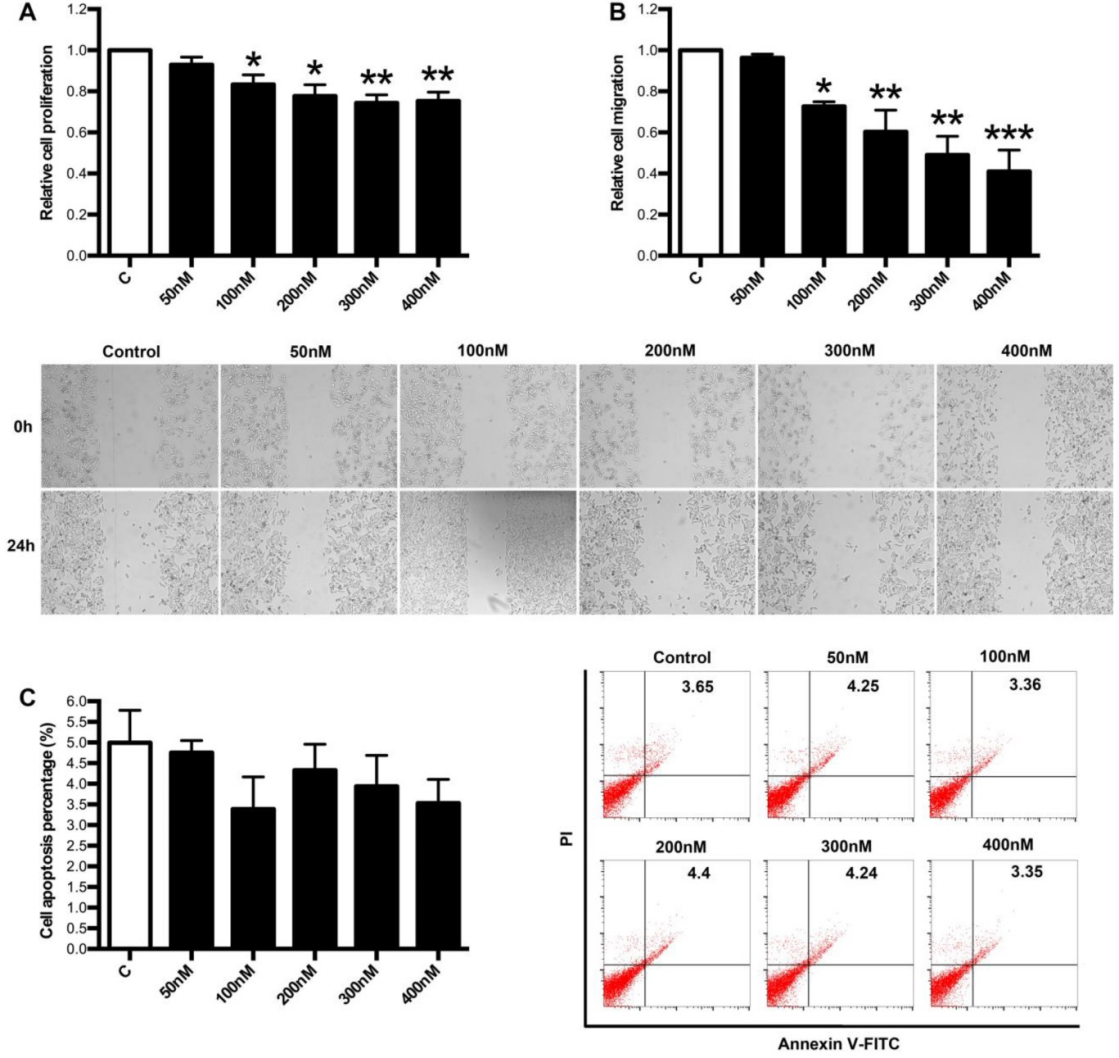
** The effects of LXA_4_ on proliferation, migration and apoptosis of colorectal cancer cells.** Colorectal cancer cell line SW480 was treated with indicated concentration of LXA_4_ for 24 h. Cell proliferation, migration and apoptosis were detected by using MTT, wound healing assay and flow cytometry respectively. (A) Effect of LXA_4_ on SW480 proliferation. (B) Effect of LXA_4_ on SW480 migration. (C) Effect of LXA_4_ on SW480 apoptosis. Results are expressed as means±SEM from three independent experiments. *P<0.05, **P<0.01 and ***P<0.01 versus control group, two-tailed Student's t-test

Next, we focused on the effects of LXA_4_ on proliferation, apoptosis and migration of colorectal cancer cells by employing human colorectal cancer cell line SW480. As shown in Fig. 6A, 100, 200, 300, 400 nM of LXA_4_ could inhibit the proliferation of SW480 cells, although 50 nM of LXA_4_ had no effect on SW480 proliferation. Moreover, 100, 200, 300, 400 nM of LXA_4_ could effectively inhibit the migration of SW480 cells, while 50 nM of LXA_4_ had no obvious effect on SW480 migration (Fig. 6B). However, LXA_4_ has no significant effect on the apoptosis of SW480 cells (Fig. 6C). These results suggest that LXA_4_ could inhibit the growth and migration of colorectal cancer cells *in vitro*.

### Regulation of LXA_4_ on proliferation, apoptosis and migration of ACM-stimulated colorectal cancer cells

**Figure 7:**
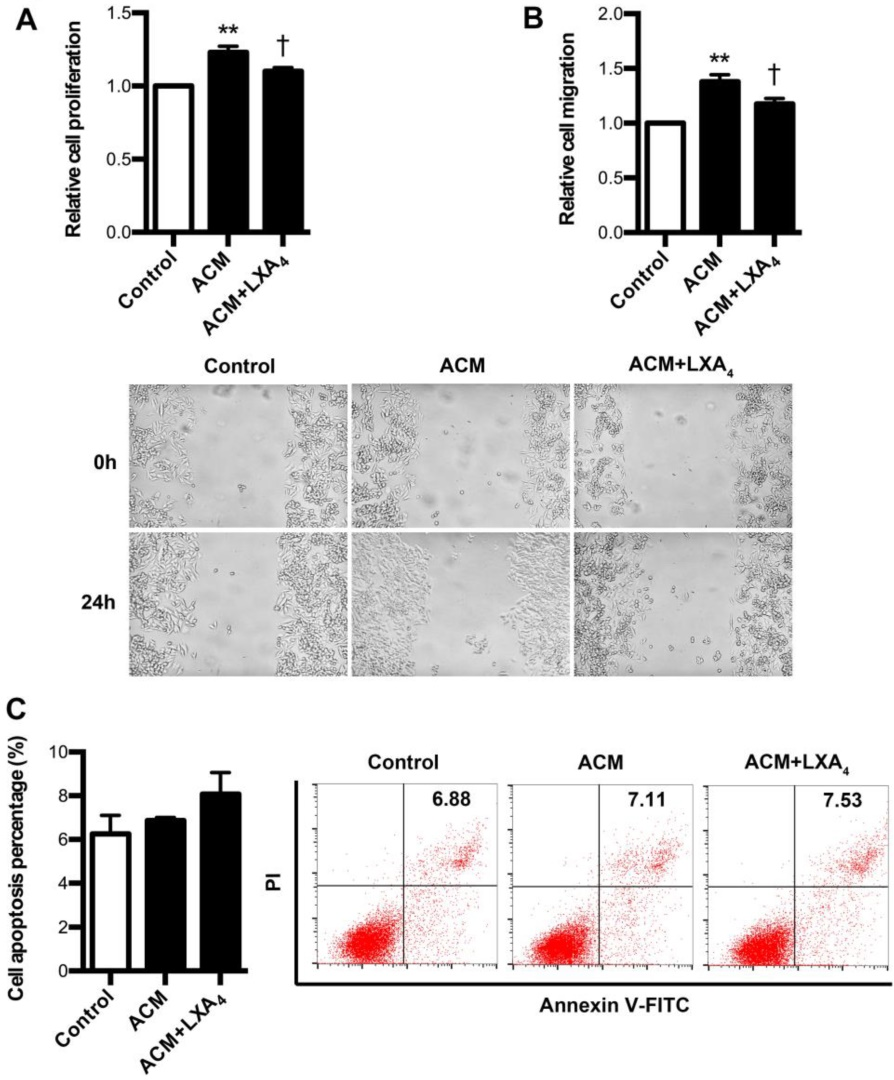
** The effects of LXA_4_ on the proliferation, migration and apoptosis of ACM-stimulated colorectal cancer cells.** Human peripheral monocytes were prepared and treated with blank, LPS and LPS+LXA_4_ respectively. Then, different CM was collected and added into the medium of SW480 cells. After 24 h, cell proliferation, migration and apoptosis were detected by using MTT, wound healing assay and flow cytometry respectively. (A) Effect of LXA_4_ on SW480 proliferation stimulated by ACM. (B) Effect of LXA_4_ on SW480 migration stimulated by ACM. (C) Effect of LXA_4_ on SW480 apoptosis stimulated by ACM. Results are expressed as means±SEM from three independent experiments. **p<0.01 versus control group, ^†^P<0.05 versus ACM group, one-way ANOVA with S-N-K posttest

Since it had been confirmed that tumor-associated macrophages (TAMs) were found to be critical parts of tumor immune microenvironment, we further explore whether LXA_4_ could affect proliferation, apoptosis and migration of colorectal cancer cells remodeled by ACM. To verify this, ACM was collected from LPS-treated human peripheral blood monocytes. As shown in Fig. 7A, SW480 proliferation was obviously increased by ACM, while this promotion was inhibited by LXA_4_. Similar results were also obtained in migration (Fig. 7B). However, LXA_4_ has no significant effect on the apoptosis of ACM-stimulated SW480 cells (Fig. 7C). These results suggest that LXA_4_ could antagonize the proliferation and migration of colorectal cancer cells stimulated by ACM.

## Discussion

In the present study, we demonstrated for the first time that LXA_4_ could suppress early colorectal cancer development via regulating tumor-associated inflammation and immune microenvironment. Several implications may be drawn from the current study: 1) colorectal cancer development is associated with a deficiency of LXA_4_, the endogenous anti-inflammatory and pro-resolving mediator. 2) LXA_4_ might act as a double-edged sword in tumor: the protumor effect by inhibiting antitumor immunity at late colorectal cancer development and the antitumor effect by preventing tumor-related inflammation at early colorectal cancer development. 3) LXA_4_ might be involved in controlling the transition from inflammation to colorectal cancer.

In the study, the LXA_4_ levels in both serum and tumor of colorectal cancer patients were decreased, implicated LXA_4_ deficiency in colorectal cancer development. Furthermore, the expressions of LXA_4_-synthesizing enzymes ALOX15 and ALOX15B were down-regulated in colorectal cancer. It's has been showed that metabolites of ALOX15 and ALOX15B are anti-tumorigenic^4^, and their down-regulation may represent important pro-tumor factors in mediating colorectal cancer development. However, LXA_4_-synthesizing ALOX5 was strikingly up-regulated in colorectal cancer. ALOX5 is the key enzyme for the biosynthesis of both proinflammatory LTB_4_ and proresolving LXA_4_. Upon stimulation, it translocates to the nuclear membrane to form an enzyme complex, which converts AA to LTs^14^. ALOX5 activation but ALOX15 and ALOX15B inhibition suggest possible class switching from LXA_4_ to LTB_4_ production. Here, we indeed found that ALOX5 activation contributed to enhanced LTB_4_ but not LXA_4_ biosynthesis in colorectal cancer. LTB_4_ has been shown to be increased in intestinal tumors of mice model, and could stimulate the proliferation of colon cancer cells, supporting its pro-tumorigenic effect^15^. Melstrom *et.al* demonstrated that ALOX5 is overexpressed in colon cancer, and inhibition of ALOX5 could inhibit tumor growth in colon cancer xenograft model^16^. Thus, ALOX5 activation and LTB_4_ increase may represent important priming factors in prompting colorectal cancer development. In the study, ALOX12 was found to be up-regulated in colorectal cancer. It has been confirmed that increased risk of colorectal cancer was associated with ALOX12 genetic polymorphisms^17^, and ALOX12 is overexpressed in colorectal cancer^18^, supporting ALOX12 as another important pro-tumorigenic factor in colorectal cancer development. Additionally, colorectal polyp and tumor tissue show progressively more intense and diffuse staining for ALOX5 and ALOX12 compared with surrounding tissue, indicating that ALOX5 and ALOX12 expression is activated early in colorectal cancer development and as the degree of malignancy increases, ALOX5 and ALOX12 expression becomes more intense and diffuse in distribution. Hence, the dysregulation of LXA_4_-synthesizing enzymes may be the reason for LXA_4_ deficiency in colorectal cancer, which may represent important pro-tumor factors in mediating colorectal cancer development.

A hallmark of cancer-associated inflammation is the presence of immune cells and inflammatory mediators in tumor microenvironment that can lead to tumor growth, progression and metastasis^1^. In the study, we observed obvious immune cells infiltration into tumor. Immune cells that infiltrate in tumor engage in an extensive and dynamic crosstalk with cancer cells, playing decisive roles at different stages of tumor development, including initiation, promotion, malignant conversion, invasion, and metastasis^1^. As major players in connecting inflammation and cancer, TAMs summarize a number of functions, which ultimately have an important impact on tumor progression 19. Here, we also showed enhanced macrophages infiltration into tumor, suggesting critical roles of TAMs in colorectal cancer. Moreover, we focused on the expressions of inflammatory mediators in colorectal cancer, since proinflammatory mediators are implicated in the pathogenesis of colon carcinogenesis 4. We showed here higher serum levels of IL1B, IL6, CXCL8, TNFA, CCL2 and tumor levels of IL1B, IL6, CXCL8, TNFA in colorectal cancer, suggesting the involvement of these inflammatory factors in colorectal cancer. Taken together, these clinical results suggest that colorectal cancer development is correlated with tumor immune microenvironment.

By employing subcutaneous xenograft mice model, we found that LXA_4_ played different roles at different stages of tumor growth: inhibiting early while promoting late tumor growth. We showed previously that Tregs depletion in established large tumors resulted in the production of LXA_4_ and the latter consequently induced the generation of MDSCs, thus promoting tumor growth^20^, suggesting that the interplay among Tregs, MDSCs, and LXA_4_ tunes the regulation of tumor growth. Thus, colorectal cancer development was differentially regulated by LXA_4_, with its important inhibitory roles at early stage of transition from inflammation to colorectal cancer.

In an attempt to interpretation of the differential effects of LXA_4_ on colorectal cancer, we investigated the influence of LXA_4_ on peripheral immune cells in subcutaneous xenograft mice model. Interestingly, LXA_4_ could up-regulate the percentage of neutrophil in peripheral leukocytes, while down-regulate lymphocytes and its percentage at late tumor growth. However, LXA_4_ could down-regulate neutrophils and its percentage, up-regulate lymphocyte percentage and down-regulate the counts and percentage of monocyte at early tumor growth. Riesco reported that a positive significant correlation is found between cancer curability and the total number of peripheral lymphocytes; a negative correlation is found between the total number of peripheral neutrophils and cancer curability; no correlation is found between curability of cancer and monocytes, indicating that the immunologic activity of peripheral lymphocytes may be a favorable factor in the cure of cancer 21. Moreover, Rao *et.al* reported that increased intratumoral neutrophil in colorectal carcinomas correlates closely with malignant phenotype and predicts patients' adverse prognosis^22^. Indeed, neutrophils can have significant effects on the tumor microenvironment by production of cytokines, chemokines, reactive oxygen species and proteinases, which influence inflammatory cell recruitment and activation, and regulate tumor cell proliferation, angiogenesis and metastasis^23^. Here, our results suggest that LXA_4_ may inhibit early while prompt late tumor growth through differentially modulating peripheral immune cells.

Since MAPK and NF-κB pathways are important signals in mediating tumorigenesis, including tumor growth and tumor-associated inflammation^13^, ^24^, ^25^, we examined the expressions of ERK, P38 and NF-κB in mice subcutaneous xenograft. Interestingly, LXA_4_ treatment could obviously suppress p-ERK, p-P38 and NF-κB, suggesting that LXA_4_ may inhibit colorectal cancer and associated inflammation via modulating ERK, P38 and NF-κB pathways.

The epithelial-mesenchymal transition (EMT) is a highly conserved cellular program that allows polarized, immotile epithelial cells to convert to motile mesenchymal cells. This important process was initially recognized during several critical stages of embryonic development and has more recently been implicated in promoting carcinoma invasion and metastasis 26. To examine the direct roles of LXA_4_ on colorectal cancer cell development *in vitro*, we studied the effects of LXA_4_ on proliferation, migration and apoptosis of colorectal cancer cell line SW480. As expected, LXA_4_ could effectively inhibit the proliferation and migration of SW480, although it has no significant effect on SW480 apoptosis. These results suggest that LXA_4_ could inhibit colorectal cancer via interfering tumor cell development. Meanwhile, we also explored the effects of LXA_4_ on proliferation, migration and apoptosis of colorectal cancer cells stimulated by ACM, since TAM, a major player in the connection between inflammation and cancer, summarizes a number of functions (e.g., promotion of tumor cell proliferation and angiogenesis) during tumor progression^19^. Importantly, SW480 cell proliferation and migration was obviously increased by ACM, while this promotion was inhibited by LXA_4_. Our results suggest that LXA_4_ could suppress colorectal cancer cell development *in vitro*.

In conclusion, LXA_4_ deficiency may result in colorectal cancer development, which might be ascribed to a dysregulation in tumor immune microenvironment, providing new insights into the pathogenesis of colorectal cancer. Our data in mice model show that LXA_4_ plays opposite roles in tumor development: inhibiting early while promoting late tumor growth, which might be explained by the results that LXA_4_ promotes anti-tumor peripheral immune environment and remodels tumor immune microenvironment at early tumor growth but contributes to pro-tumor peripheral immune environment at late tumor growth. This study proposes that LXA_4_ suppress early colorectal cancer development via regulating tumor immune microenvironment, providing new insights into the understanding of colorectal cancer. Our data suggests that LXA_4_ administration might be a potential strategy against early colorectal cancer development, and also contribute to develop other potential therapeutic strategies by targeting immune microenvironment in preventing colorectal cancer.

## Supplementary Material

Supplementary figures.
