# Supplementary material for: Colorectal Cancer Is Associated with a Deficiency of Lipoxin A4, an Endogenous Anti-inflammatory Mediator
Source: J Cancer. 2019 Aug 20;10(19):4719–30. doi: 10.7150/jca.32456 (PMC6746129; doi:10.7150/jca.32456)
Supplement: Supplementary file 1 — Supplementary figures. [file jcav10p4719s1.pdf]

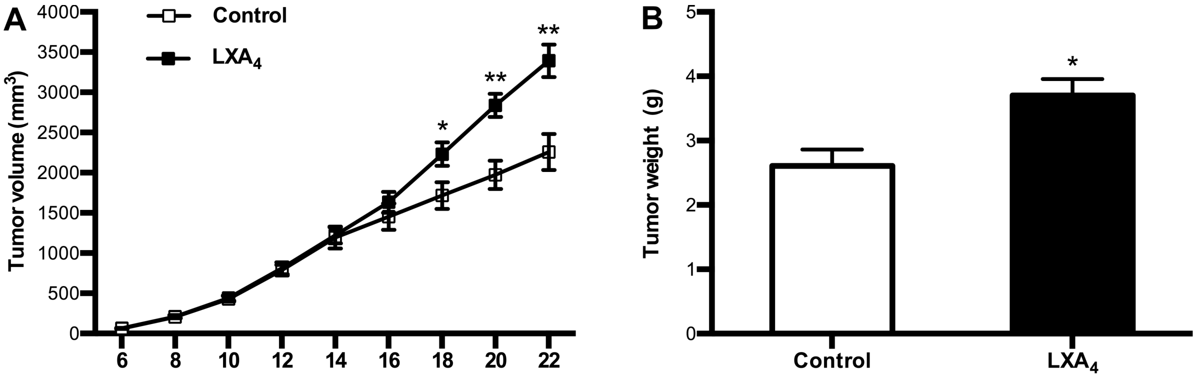

Fig. S1. The promotion of LXA_4 on late subcutaneous xenograft in mice. Mouse CRC cell line CT26 was used to prepare subcutaneous xenograft. Mice were injected *i.p.* with LXA_4 on day 15 after inoculation. The volume of the peripheral tumor was measured every other day before and after inoculation. At the time of autopsy on day 22, tumors were dissected and weighed. (A) Comparison of tumor volume between control and LXA_4 group. (B) Comparison of tumor weight between control and LXA_4 group. Results are expressed as means±SEM (n=7 mice in each group). *p<0.05 and **p<0.01 versus control group, two-tailed Student's t-test.

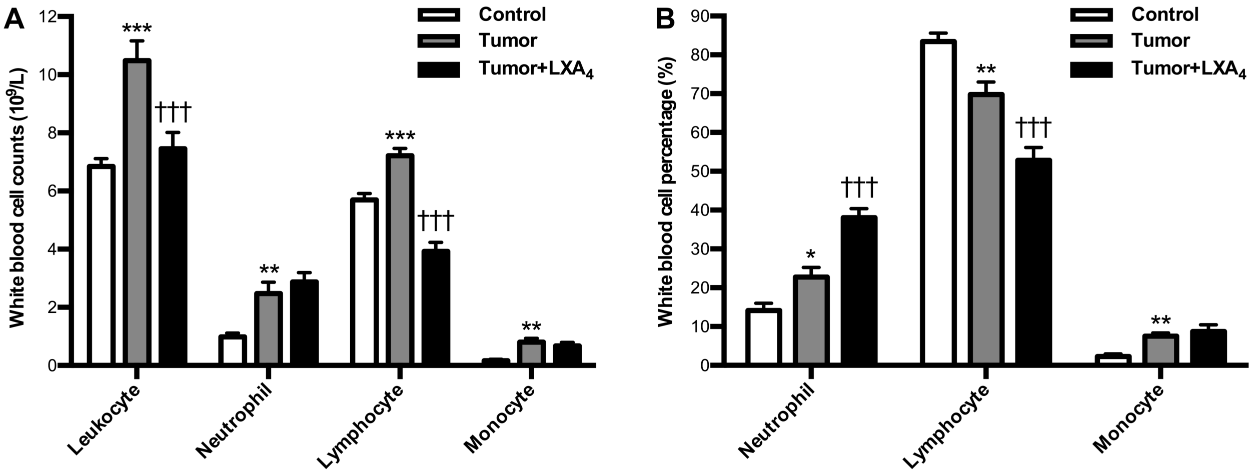

Fig. S2. The regulation of LXA_4 on peripheral immune cells in late subcutaneous xenograft mice model. Subcutaneous xenograft was prepared, and mice were injected *i.p.* with LXA_4 on day 15

after inoculation. At the time of autopsy on day 22, peripheral blood was collected from mouse eyes, and peripheral leukocytes were classified and counted by whole blood cell counter. (A) Comparison of peripheral blood leukocyte counts between control, tumor and LXA_4 group. (B) Comparison of peripheral blood leukocyte classification between control, tumor and LXA_4 group. Results are expressed as means±SEM (n=7 mice in each group). *$p<0.05$, **$p<0.01$ and ***$p<0.001$ versus control group, $^{\dagger\dagger\dagger}P<0.001$ versus tumor group, one-way ANOVA with S-N-K posttest.
